# A meta-analysis of the diagnostic value of microRNA-1246 for malignant tumors

**DOI:** 10.1097/MD.0000000000015848

**Published:** 2019-05-31

**Authors:** Chunlin Xie, Tao Huang, Zhaowei Teng, Shuanglan Xu, Junhui Bu, Mengzhou Li, Yibing Zhang, Jing Zhang

**Affiliations:** a Department of Thoracic Surgery, The 1st Affiliated Hospital of Kunming Medical University, Kunming; b Department of Orthopaedics, The 6th Affiliated Hospital of Kunming Medical University, Yuxi; c Department of Respiratory Medicine, The Fourth Affiliated Hospital of Kunming Medical University, The Second People's Hospital of Yunnan Province, Kunming, Yunnan, PR China.

**Keywords:** cancer, diagnostic value, meta-analysis, microRNA-1246

## Abstract

**Background:**

Cancer morbidity and mortality are growing rapidly worldwide. There have been an increasing number of studies on the correlation between miRNA1246 expression in circulating blood and tumors; however, no comprehensive conclusion has been reached. Therefore, this meta-analysis was carried out to systematically evaluate the diagnostic value of blood levels of microRNA-1246 for malignant tumors.

**Methods:**

We searched PubMed, MEDLINE, Embase, The Cochrane Library, the China National Knowledge Internet (CNKI), and Wanfang databases from the inception of each database until November 2018. The quality of the included literature was evaluated using the quality assessment tool called Quality Assessment of Diagnostic Accuracy Studies-2 (QUADAS-2). The data were pooled using Stata14 and Meta-DiSc 1.4 software.

**Results:**

Seven studies were included. The pooled sensitivity (SENS) was 0.80 (95% CI 0.65–0.89), the pooled specificity (SPEC) was 0.77 (95% CI 0.70–0.83), the pooled positive likelihood ratio (PLR) was 3.55 (95% CI 2.53–4.99), the pooled negative likelihood ratio (NLR) was 0.26 (95% CI 0.16–0.47), the pooled diagnostic odds ratio (DOR) was 13.78 (95% CI 5.84–32.5), and the area under the curve (AUC) was 0.83 (95% CI 0.79–0.86). The result of Deeks’ funnel plot was *P* = 0.31, indicating a lack of publication bias.

**Conclusion:**

MicroRNA-1246 in the blood can be used as a good indicator for the diagnosis of malignant tumor diseases and has a moderate diagnostic accuracy for the differentiation of patients with malignant tumors from healthy people.

## Abstract 2

Supplemental Digital Content is available in the text

## 1 Introduction

Cancer morbidity and mortality are growing rapidly worldwide. In 2018, 18.1 million cancer patients were diagnosed worldwide, and 9.6 million patients died of cancer.^[1,2]^ In recent years, there have been an increasing number of studies on the correlation between miRNA expression and tumors. Many studies have shown that microRNAs are widely involved in the occurrence and development of malignant tumors and thus can be used as molecular markers or potential therapeutic targets for the diagnosis and prognosis of cancers.^[3–5]^

MicroRNAs are a diverse family of molecules composed of noncoding sequences that are 19–25 nucleotides long.^[6]^ Mature microRNAs can be directed to the 3’ end of their target mRNA by base pairing, which can result in the decreased stability of the target mRNA and the inhibition of its translation.^[7–9]^ The biological functions of multiple microRNAs have been investigated using microRNA knockout models and transgene overexpression assays, for example, in tumors, microRNAs can affect tumor cell growth, proliferation, invasion, and apoptosis.^[10–14]^ Studies have found that different types of microRNAs have different expression levels in the same malignant tumor, and the same microRNA is expressed differently in different types of malignant tumors.^[15,16]^ A study reported that the expressions of micro-382-3p and micro-1246 in the serum of breast cancer patients were up-regulated compared with those in the healthy group. However, the expressions of micro-598-3p and micro-184 in the serum of breast cancer patients were down-regulated compared with those in the healthy group. Based on such an observation, a conclusion that different microRNAs have different expressions in the same malignant tumor disease can be achieved.^[17]^ Another study reported that the expression of microRNA-451 was up-regulated in the serum of patients with esophagus cancer and breast cancer, while it was down-regulated in the serum of patients with renal carcinoma. This indicates that the expression levels of the same microRNA in different types of malignant tumor are different.^[18]^ Like the above situation, we have also counted several other studies,^[18–25]^ as shown in Supplementary Tables. I and II.

In recent years, microRNA-1246 has attracted the attention of many researchers, and many of them have demonstrated that microRNA-1246 acts as a proto-oncogene in many cancers.^[26,27]^ In the human genome, the microRNA-1246 gene is located on the second chromosome (2q31.1) and is a transcriptional target of p53 that is involved in the regulation of the known functions of p53, including in the cell cycle, apoptosis and senescence.^[28]^ Studies have shown that in the processes involved in the development of breast cancer, colon cancer, oral squamous cell carcinoma, pancreatic cholangiocarcinoma, lung cancer, pancreatic cancer, cervical cancer, ovarian cancer, and gastric cancer,^[4,27,29–34]^ cancer cell proliferation, invasion and metastasis are regulated by microRNA-1246. Multiple studies have used noninvasive methods to acquire miRNA1246 from the plasma or serum samples of cancer patients and detected abnormal expression of microRNA-1246,^[17,34–39]^ demonstrating that the detection of changes in the content of microRNA-1246 in cancer patients can provide some reference value for the diagnosis of certain malignant tumor diseases. There have been an increasing number of studies on the correlation between miRNA1246 expression in circulating blood and tumors; however, due to the small sample size of individual studies and the different types of cancers investigated, no comprehensive conclusion has been reached. Seven relevant studies were included in this study, and a meta-analysis was conducted to further systematically evaluate the diagnostic value of microRNA-1246 in circulating blood for cancer.

## 2 Materials and methods

### 2.1 Search strategy

To identify eligible studies for this systematic review, the 2 investigators searched in the PubMed, MEDLINE, Embase, The Cochrane Library, the China National Knowledge Internet (CNKI), and Wanfang databases from the inception of each database until November 2018. All analyses were based on previous published studies and thus no ethical approval and patient consent are required. The search terms included “(cancer OR tumor OR squamous cell carcinoma OR adenocarcinoma)” AND “(microRNA1246 OR miRNA1246 OR miR1246)” AND “(diagnostic accuracy OR sensitivity OR specificity OR AUC).” A search strategy, in accordance with a professional search process, was determined, which was used to search relevant literature. Meanwhile, we manually searched for relevant studies and reviewed the references included in the identified articles.

### 2.2 Inclusion and exclusion criteria

Studies were included that met the following criteria: the diagnostic value for cancer of microRNA-1246 content in the serum or plasma was evaluated; the data in the study could be converted into a 2 × 2 table; the diagnoses of malignant tumors were histopathologically confirmed, and the control group was composed of healthy people; and the study was published in Chinese or English. The exclusion criteria were as follows: repeated studies; articles that were reviews, lectures, case reports, etc.; studies that did not provide intact information or data; and animal experiments and other articles that were not relevant to this study.

### 2.3 Data extraction

Two researchers (BJH and LMZ) independently extracted the required data from the eligible studies and then assessed the extracted data. Any disagreement between the 2 researchers was resolved by reaching a consensus that was determined by the third researcher (ZYB) through discussion. Finally, the studies meeting all the criteria were included in the meta-analysis.

### 2.4 Quality assessment

The quality of the included studies was assessed using the Quality Assessment of Diagnostic Accuracy Studies-2 (QUADAS-2) tool. Each question in the assessment was scored as “yes,” “no” or “unclear.”^[40]^ In this assessment, there are 14 items in 4 parts, namely, the selection of the case, the index test, the reference standard and the case process, and the progress. The scores are given as “Yes (1),” “No (−1)” and “Unclear (0).” Finally, the total score represents the quality of the study as follows: 0–7 indicates low-quality literature with a high possibility of bias, while 8–14 indicates high-quality literature.

### 2.5 Statistical analysis

Data analysis was performed using Meta-Disc1.4 and Stata14 software. I^2^ values, P values and bivariate boxplot maps, were used to test for heterogeneity. If there was no significant heterogeneity between the studies (*P* **>** 0.1, *I*^2^≤50%), a fixed effects model was used; if there was significant heterogeneity between the studies (*P***≤**0.1, *I*^2^ > 50%), a random effects model was used. Additionally, a receiver operating characteristic curve (ROC) was drawn. Spearman's rank correlation coefficients for the log of sensitivity and the log of (1-specificity) were calculated, which were used to investigate whether there was a threshold effect. If there was a threshold effect, it was evaluated whether the heterogeneity was caused by it. If there was heterogeneity caused by nonthreshold effects, meta-regression was used to determine its source. SENS, SPEC, PLR, NLR, DOR, and the corresponding 95% CIs were pooled, the SROC curve was drawn, and the AUC was calculated. On this basis, the diagnostic value of microRNA-1246 for malignant tumors could then be evaluated, and the data obtained from the bivariate model were validated using the Hierarchical Summary Receive Operating Characteristic (HSROC) model. A sensitivity analysis, in which the included studies were removed one by one, was conducted to assess the impacts of individual studies. A Fagan diagram was drawn, which was used to illustrate the relationship between pretest probability, likelihood ratio, and post-test probability. Deeks’ funnel plot was used to evaluate whether publication bias existed.^[41]^

## 3 Results

### 3.1 Literature search

**Figure 1:**
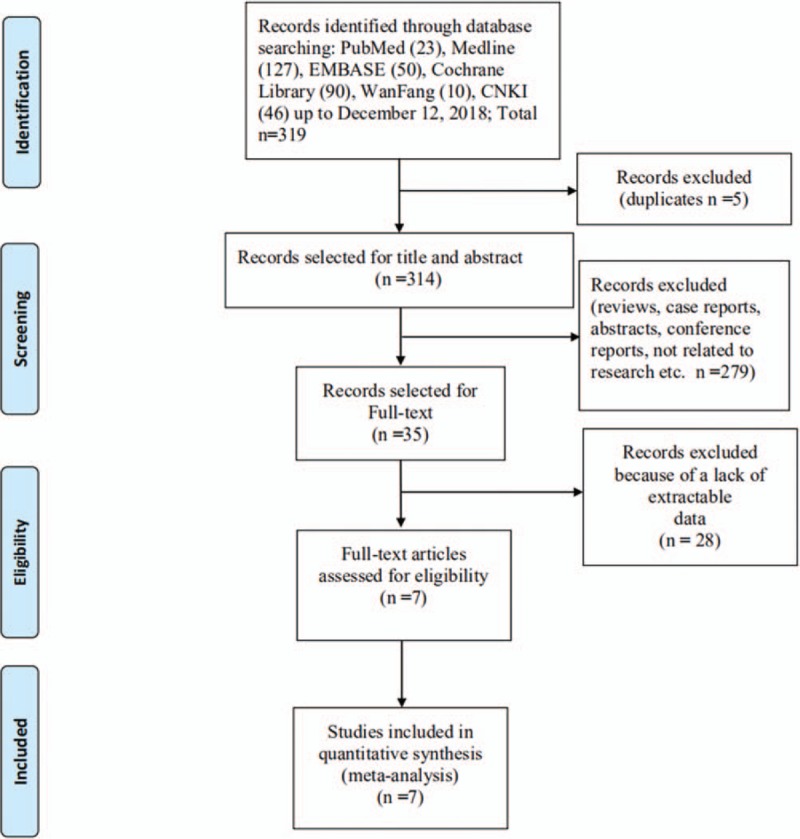
Literature screening process and results.

The process of selecting the eligible studies is shown in Figure 1. First, the studies obtained by searching the databases were PubMed n = 23, MEDLINE n = 127, Embase n = 50, Ovid n = 61, Cochrane Library n = 90, Wanfang n = 10, CNKI n = 46, a total of 319 articles. Excluding 5 duplicate articles, the final preliminary search results were 314 articles. Second, based reading the titles and abstracts of the 314 articles that were obtained, 279 studies that were not relevant were excluded, including literature reviews, abstracts, case reports, and conference reports. The preliminary screening resulted in 35 studies. Finally, reading the full text articles and comparing them to inclusion criteria, the studies without extractable data, and 7 studies were finally enrolled.

### 3.2 Characteristics of the eligible studies

In this study, 7 articles (6 English and 1 Chinese) were included, representing 7 independent case-control experiments (2 in breast cancer, 2 in colorectal cancer, 1 in oesophageal cancer, 1 in ovarian cancer, and 1 in liver cancer), involving a total of 975 subjects. The 7 case–control experiments were performed in China (n = 3), Japan (n = 2), and Italy (n = 2). Serum (n = 6) and plasma (n = 1) samples were collected from which microRNA was extracted via reverse transcription-quantitative polymerase chain reaction (RT-PCR). The basic characteristics of the studies are shown in Table 1.

**Table 1:**
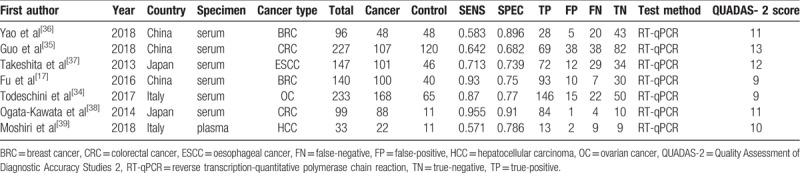
Summary of information from the included studies.

### 3.3 Assessment of study quality

The quality of the included literature was assessed using QUADAS-2. The results are presented in Table 2. The QUADAS-2 score ranges between 9 and 13 points, indicating that all studies are high quality, ensuring the reliability of the meta-analysis results.

**Table 2:**
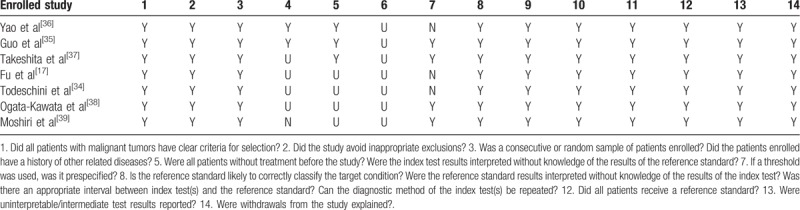
Quality assessment of 7 diagnostic accuracy studies.

### 3.4 Data analysis

**Figure 2:**
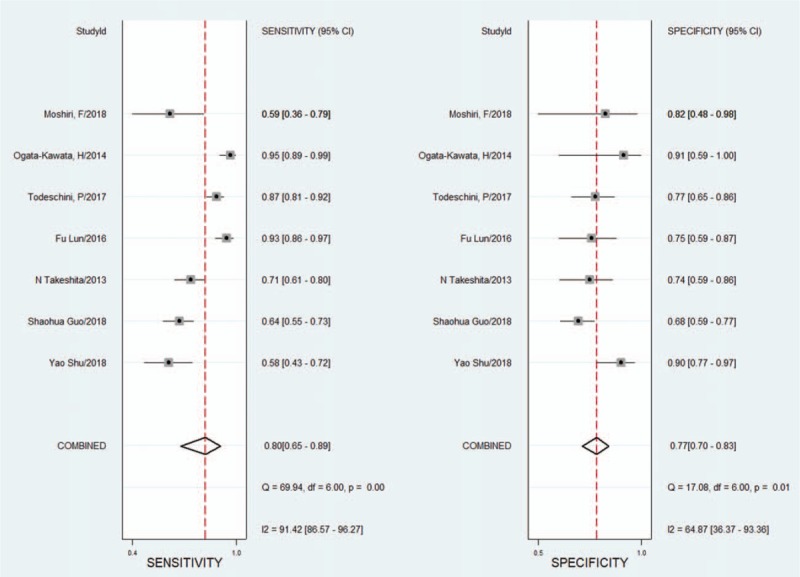
Forest plots of the pooled sensitivity and specificity results for the 7 included studies. CI = confidence interval, DF = degrees of freedom, *I*^2^ = inconsistency index, Q = Cochran's *Q* value.

The sensitivity and specificity of the 7 included studies were pooled and analysed. From the forest map (Fig. 2) of the pooled SENS and SPEC, it was found that the *I*^2^ values of the pooled SENS and SPEC were 91.42 (86.57–96.27) and 64.87 (36.37–93.36), respectively. The results indicated that heterogeneity existed among the included studies. Thus, the meta-analysis was performed using a random effects model.

**Figure 3:**
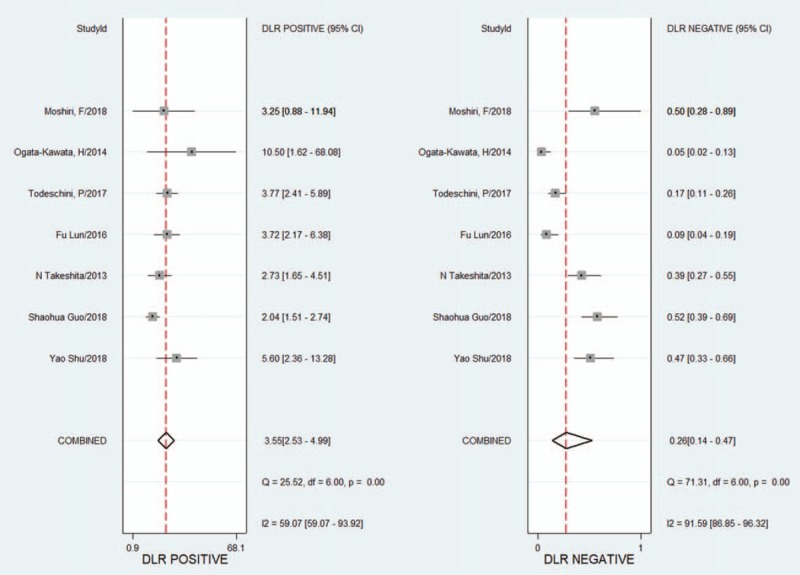
Forest plots of the positive and negative likelihood ratios for microRNA-1246 in the diagnosis of cancer. CI = confidence interval, DF = degrees of freedom, DLR = diagnostic likelihood ratio, *I*^2^ = inconsistency index, Q = Cochran's *Q* value.

**Figure 4:**
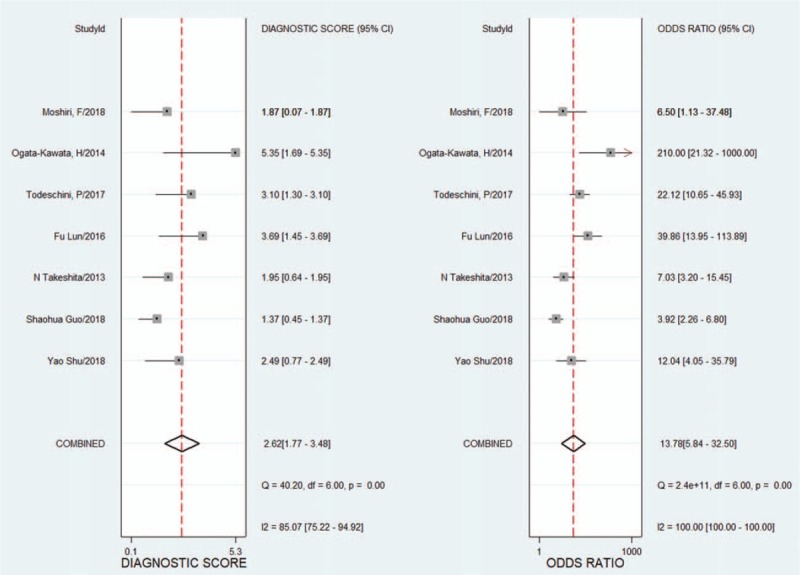
Forest plots of the pooled diagnostic odds ratio for microRNA-1246 in the diagnosis of cancer. CI = confidence interval, DF = degrees of freedom, *I*^2^ = inconsistency index, Q = Cochran's *Q* value.

**Figure 5:**
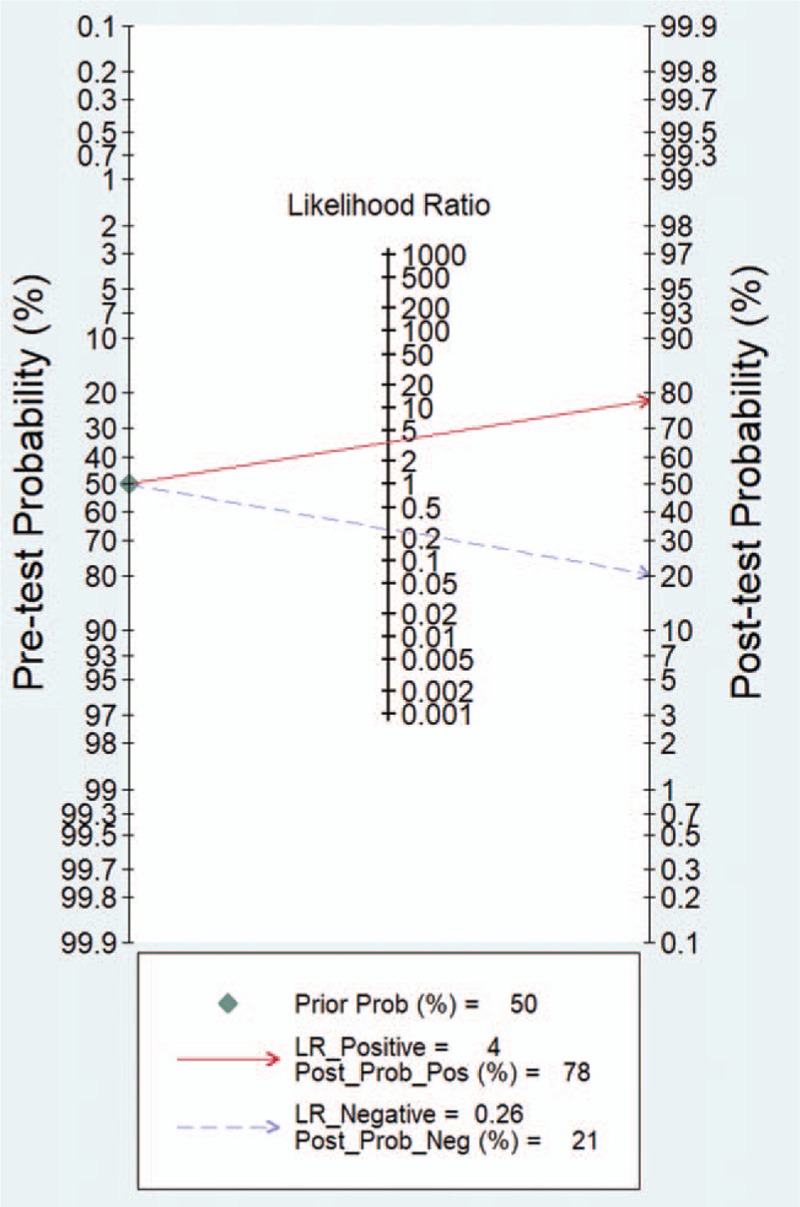
Fagan's nomogram for assessing the post-test probabilities. LR = likelihood ratio.

Because of the heterogeneity caused by the nonthreshold effect among the studies, the random effects model was used to pool the SENS, SPEC, PLR, NLR, and DOR of the included studies. The pooled SENS was 0.80 (95% CI 0.65–0.89) (see Fig. 2), the pooled SPEC was 0.77 (95% CI 0.70–0.83) (see Fig. 2), the pooled PLR was 3.55 (95% CI 2.53–4.99) (see Fig. 3), the pooled NLR was 0.26 (95% CI 0.16–0.47) (see Fig. 3), and the pooled DOR was 13.78 (95% CI 5.84–32.5) (see Fig. 4). It is assumed that the pretest probability of cancer was 0.5 (that is, before detecting microRNA-1246 in serum or plasma, the probability of patients suffering from malignant tumors is 0.5.). Then, the Fagan plot (Fig. 5) was generated. As shown in the plot, the positive result improved the post-test probability of suffering from cancer to 78%, while the negative result dropped that of having cancer to 21%. The PLR was 4, and the NLR was 0.26.

**Figure 6:**
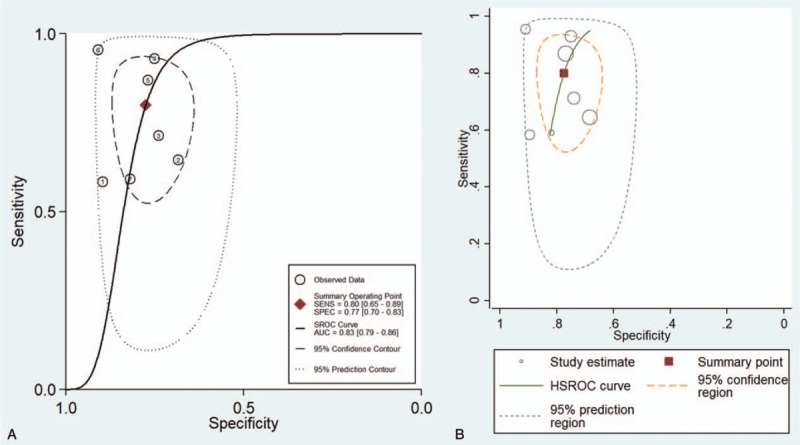
(A) SROC curve with the pooled estimates of sensitivity, specificity and area under the curve. AUC = area under the curve, SENS = sensitivity, SPEC = specificity. (B) HSROC curve for microRNA-1246 in the diagnosis of cancer. HSROC = hierarchical summary operating characteristic, SROC = summary receiver operating characteristic.

The SROC curve illustrates the performance of the overall test and balances its sensitivity and specificity. As described in the drawn SROC curve (Fig. 6A), 5 studies were within the 95% CI, and the other 2 studies were within the 95% predictable range. The optimal cut-off point had a SENS of 0.80 (0.65–0.89) and a SPEC of 0.77 (0.70–0.83). Additionally, the AUC was 0.83 (0.79–0.86). In the results given by the HSROC model (Fig. 6B), the β (beta) estimation and the 95% CI were −1.22 (95% CI − 2.70–0.26), and *z* = −1.62, *P* = .106 (*P* > .5). The λ (lambda) estimation and the 95% CI were 3.03 (95% CI 1.84–4.21).

### 3.5 Threshold effect and heterogeneity

**Figure 7:**
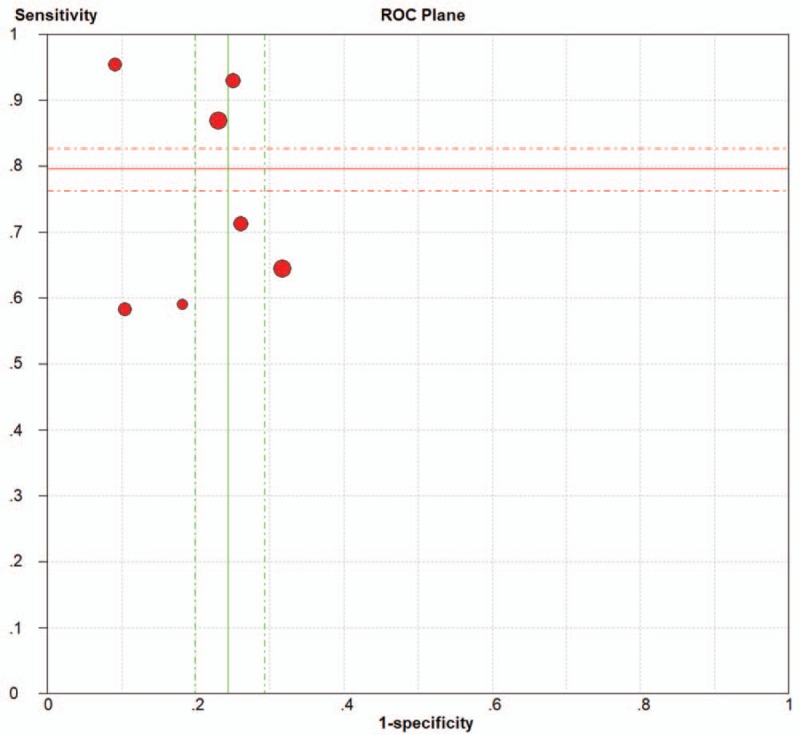
ROC space for the assessment of the threshold effect of microRNA-1246.

In diagnostic clinical trials, the causes of heterogeneity between studies include threshold effects and nonthreshold effects. Threshold effects are partly caused by the disparity between the sensitivity and specificity, and the SENS and SPEC of the Spearman's correlation coefficient are often used to evaluate threshold effects.^[42]^ When heterogeneity between studies is caused by a threshold effect, the SENS and SPEC re negatively correlated, and the included studies show a “shoulder arm” distribution on the ROC chart. In this paper, the heterogeneity sources in the included studies were analysed, and the ROC curve was drawn (Fig. 7). However, the chart of the ROC curve did not show a “shoulder arm” point distribution. The Spearman rank correlation coefficient analysis result was *r* = −0.071, *P = *.879 (*P > 0*.05). These results indicated that heterogeneity was not caused by the threshold effect. It can be seen from the forest map (Fig. 4) obtained from the pooled DOR that the pooled DOR = 13.78 (5.84–32.5). It can also be seen that the DOR of each study and the pooled DOR are not distributed along the same line, indicating that the heterogeneity is caused by nonthreshold effects. Given that the heterogeneity was due to nonthreshold effects between studies, meta-regression analysis was performed to identify the sources of heterogeneity based on the country, cancer type, sample size, and research quality. The meta-analysis results indicate that the type of cancer and the number of samples are sources of heterogeneity (see Table 3).

**Table 3:**
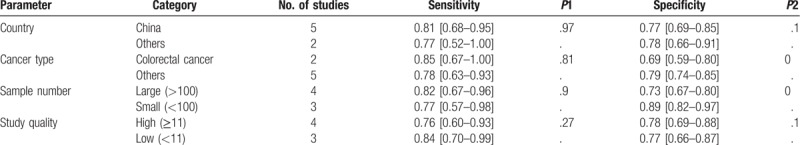
Meta-regression of 7 diagnostic accuracy studies.

### 3.6 Sensitivity analysis

**Figure 8:**
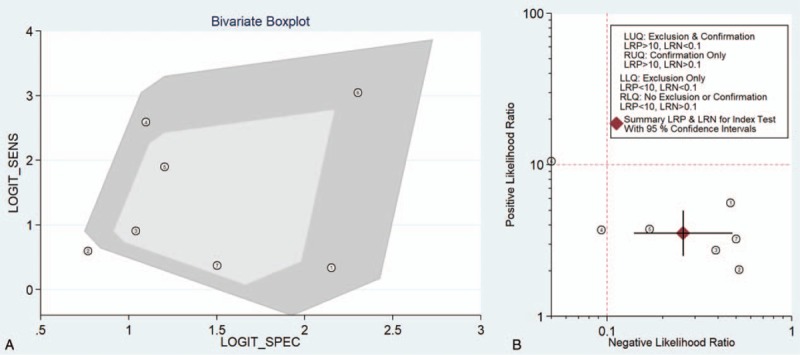
(A) Contour plot for SENS/SPEC (B) Matrix plot for PLR/NLR. NLR = negative likelihood ratio, PLR = positive likelihood ratio.

Through the bivariate boxplot (Fig. 8A) and matrix plot for PLR/NLR (Fig. 8B), 2 outlier studies ^[35,38]^ were identified and excluded. Next, the effect sizes of the remaining 5 studies were pooled; therefore, the outliers had less impact on the results of this meta-analysis (see Table 4). There was heterogeneity among the studies, and meta-regression analysis was performed to find the sources of heterogeneity sources based on the country, cancer type, sample size, and research quality. The meta-analysis results indicate that the type of cancer and the number of samples are sources of heterogeneity (see Table 4). The sensitivity analysis was used to eliminate each eligible study in turn and to evaluate any changes in the overall results to ensure the reliability and stability of the meta-analysis. In other words, in this analysis, the 7 included studies were removed one by one, and then the SENS, SPEC, PLR, NLR, and DOR of the included literature were pooled. If the above indicators showed no significant changes, the results of the meta-analysis were stable.

**Table 4:**
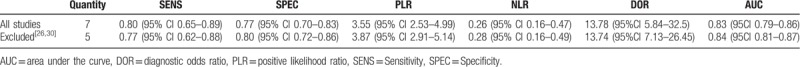
Quality assessment of 7 diagnostic accuracy studies.

### 3.7 Publication bias

**Figure 9:**
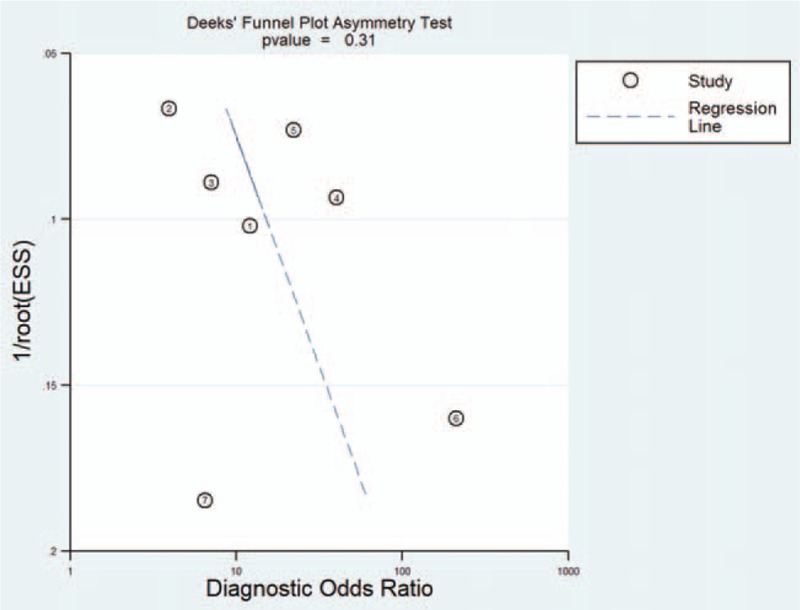
Graph of Deeks’ funnel plot asymmetry test. ESS = effective sample size.

As shown in Figure 9, linear regression was used to test the asymmetry of the funnel plot to evaluate publication bias. As depicted in Figure 9, *P* = .31, indicating that there was no obvious asymmetry in the funnel plot. The smaller the angle between the regression line and the DOR axis, the closer it is to 90° and the lower the possibility it indicates a bias. The angle in the figure is very close to 90°, indicating no significant publication bias, indicating that the results of this meta-analysis are reliable.

## 4 Discussion

The current reference standard for cancer diagnosis is still tissue biopsy. However, due to the limitations of invasive examination and the inability of some patients to accept or tolerate invasive diagnostic methods, identifying reliable tumor markers in the blood has become a new priority for many researchers. A reliable tumor marker not only reflects the early presence of tumors but also suggests the state and dynamics of advanced tumors.^[43]^ To date, many studies on the correlation between the level of microRNA-1246 in circulating blood and tumor characteristics have been conducted. The results indicate that microRNA-1246 regulates the proliferation, invasion and metastasis of cancer cells in the development of various cancers. This study is the first meta-analysis of the diagnostic value of blood levels of microRNA-1246 for cancer.

In this meta-analysis, the QUADAS-2 scores of the 7 included studies were all between 9 and 13, indicating that the included studies were relatively reliable. The pooled SENS was 0.80 (95% CI 0.65–0.89), the pooled SPEC was 0.77 (95% CI 0.70–0.83), the pooled PLR was 3.55 (95% CI 2.53–4.99), the pooled NLR was 0.26 (95% CI 0.16–0.47), the pooled DOR was 13.78 (95% CI 5.84–32.5), and the AUC was 0.83 (95% CI 0.79–0.86). The AUC reflects the accuracy of the diagnosis; when the AUC is between 0.5 and 0.7, the accuracy is low. When AUC is between 0.7 and 0.9, the diagnostic accuracy is moderate. When the AUC is >0.9, the diagnostic accuracy is high. In this study, the AUC was 0.83 (95% CI 0.79–0.86), indicating that microRNA1246 in the blood has a moderate diagnostic accuracy for cancer. In addition, the DOR value of the pooled SENS and SPEC was 13.78, which also demonstrates the diagnostic value of microRNA-1246 in the blood for cancer. In terms of the potential for clinical application, the results of the Fagan plot indicate that if the pretest probability is assumed to be 0.5, the probability of a patient being diagnosed with a malignant tumor is 78% after the detection of microRNA-1246 in the serum or plasma, and the probability of not being diagnosed with a malignant tumor is 21%. PLR = 4 indicates that the probability of positive results in patients with malignant tumors after the detection of microRNA-1246 in the serum or plasma is 4 times that in healthy people in the control group; NLR = 0.26 indicates that the probability of a negative result in cancer patients after the detection of microRNA-1246 in the serum or plasma is 0.26 times that in the healthy population in the control group. Both the HSROC model based on Bayes theory and the bivariate model can analyse the data through the random effects model. The HSROC model is more concise than the bivariate model, but the AUC value cannot be obtained. Therefore, both methods were used for the data analysis. The β estimation and the 95% CI were −1.22 (95% CI − 2.70–0.26) and *z* = −1.62, *P = *.16 (*P > *0.5), indicating that the SROC curve is symmetrical. The λ value is the effect index of the diagnostic ability of the diagnostic test. The λ estimation and the 95% CI were 3.03 (95% CI 1.84–4.21), indicating that the detection of microRNA-1246 in the serum or plasma has is accurate for the diagnosis of malignant tumors.

When interpreting the results of this meta-analysis, the heterogeneity among studies must be considered. There is heterogeneity among the studies included in this meta-analysis. We found through meta-regression that the type of cancer and the number of samples may be the sources of the heterogeneity.

In Deeks’ funnel plot, *P* = .31, and the *P*-value was greater than 0.10, indicating that the funnel plot had no significant asymmetry. These results indicate that the detection of microRNA-1246 in the blood can be a good indicator for the diagnosis of malignant tumor. However, more well-designed studies and larger sample sizes are needed to further verify the diagnostic accuracy of blood levels of microRNA-1246.

The shortcomings of this study are as follows: unpublished data and the data currently being studied were not included, and the included studies were limited to those in Chinese and English. This may cause publication bias in the study and have a slight impact on the final pooled results. The relevant literature was limited, and some studies had small sample sizes, which may reduce the accuracy of the analysis. There was no uniform cut-off value, and different cut-off values were used in each study.

## 5 Conclusion

In summary, the results of this meta-analysis indicate that the detection of microRNA-1246 in the blood has a moderate diagnostic accuracy for the differential diagnosis of patients with malignant tumors and healthy people and has great potential as a noninvasive biomarker for the diagnosis of cancer. While currently the detection of microRNA-1246 in the blood does not clearly diagnose malignant tumors, it can provide meaningful reference information for clinicians. Currently, the types of cancers included in the study of the diagnostic value of microRNA-1246 are limited, and some studies on microRNA-1246 used tissue samples. Therefore, in the future, more well-designed studies on the diagnosis of malignant tumors by detecting microRNA-1246 in the serum should be carried out, and the sample sizes should be larger.

## Author contributions

**Conceptualization:** Chunlin Xie.

**Data curation:** Chunlin Xie.

**Formal analysis:** Jing Zhang.

**Funding acquisition:** Tao Huang.

**Methodology:** Zhaowei Teng, Yibing Zhang.

**Resources:** Yibing Zhang.

**Software:** Chunlin Xie, Shuanglan Xu, Junhui Bu, Mengzhou Li, Yibing Zhang.

**Supervision:** Chunlin Xie.

**Validation:** Chunlin Xie.

**Visualization:** Chunlin Xie.

**Writing – original draft:** Chunlin Xie.

**Writing – review & editing:** Chunlin Xie.

## Supplementary Material

Supplemental Digital Content
