# Supplementary material for: A meta-analysis of the diagnostic value of microRNA-1246 for malignant tumors
Source: Medicine (Baltimore). 2019 May 31;98(22):e15848. doi: 10.1097/MD.0000000000015848 (PMC6708943; doi:10.1097/MD.0000000000015848)
Supplement: Supplemental Digital Content [file medi-98-e15848-s001.doc]

**Supplemental Table I. Different types of microRNAs have different expression levels in the same malignant tumor**

| Study (year) | Cancer | miRNA method | microRNA(s)  up-regulated | microRNA(s)  down-regulated |
| --- | --- | --- | --- | --- |
| Chiam (2015) | Esophageal adenocarcinoma | qRT-PCR | miR-16-5p, miR-25-3p, miR-320a, miR-15b-5p | miR-30a-5p, miR-324-5p, miR-17-5p, miR-194-5p. |
| Sohn (2015) | Hepatocellular carcinoma | qRT-PCR | miR-18a, miR-221, miR-222, miR-224 | miR-101, miR-106b, miR-122, miR-195 |
| Lun (2016) | Breast cancer | qRT-PCR | miR-382-3p 、miR-1246 | miR-598-3p，miR-184 |

**Supplemental Table II. Same microRNA is expressed differently in different types**

of malignant tumors

| Study (year) | microRNA(s) | miRNA method | Cancer  up-regulated | Cancer  down-regulated |
| --- | --- | --- | --- | --- |
| Li ZZ (2019) | microRNA-451 | qRT-PCR | Esophagus cancer,  Breast cancer | Renal carcinoma |
| Helen M. (2010); Motawi TK (2015) | microRNA-195 | qRT-PCR | Breast cancer | Colon cancer, Hepatocellular carcinoma |
| Yang R (2017); Lin HM (2014); Marchini S (2011) | microRNA-20a | qRT-PCR | Gastric cancer, Prostate cancer | Epithelial ovarian cancer, |
